# Giant Pancreatic Duct Calculi in the Common Channel Two Decades Post Choledochojejunostomy: The Synergistic Impact of Persistent Anomalous Pancreaticobiliary Union and an Elongated Blind Stump

**DOI:** 10.7759/cureus.109277

**Published:** 2026-05-20

**Authors:** Daoyu Geng, Tao Liu, Yongjun Chen, Bing Wang

**Affiliations:** 1 Department of Hepatobiliary Surgery, Danjiangkou First Hospital, Shiyan, CHN; 2 Department of General Surgery, Huangmei Hospital of Traditional Chinese Medicine (TCM), Wuhan, CHN; 3 Department of Hepatic-Biliary-Pancreatic Surgery, Tongji Hospital, Tongji Medical College, Huazhong University of Science and Technology, Wuhan, CHN

**Keywords:** anomalous pancreaticobiliary ductal union, blind stump, choledochojejunostomy, common channel, endoscopic sphincterotomy, late onset, lithotomy, pancreatic duct calculi

## Abstract

Anomalous pancreaticobiliary ductal union (APBDU) represents a congenital anomaly predisposing patients to biliary dilatation. This aberrant architecture facilitates the intermingling of pancreatic and biliary fluids, thereby elevating susceptibility to cholelithiasis and malignancies of the biliary tract. While radical cholangiojejunostomy constitutes the standard therapeutic intervention, it frequently fails to rectify the underlying APBDU. Retention of an excessively elongated common bile duct (CBD) remnant can generate a blind loop, precipitating stone formation within the common channel years following the procedure. Such delayed manifestations in adult populations remain scarcely documented. We herein present a case involving a patient with P-B-type APBDU who exhibited symptoms two decades post-cholangiojejunostomy. Radiological assessment identified a CBD stump measuring approximately 2.5 cm alongside a substantial pancreatic calculus. This instance underscores the compounding effects of uncorrected APBDU morphology and a surgically induced, overly extensive blind loop, extending beyond the conventional paradigm of pancreaticobiliary reflux. Management was achieved exclusively via endoscopic sphincterotomy coupled with accessory cholangioscopy-assisted lithotomy. This report bridges a critical knowledge gap regarding extremely late-onset pancreatic duct stones within the common channel subsequent to cholangiojejunostomy. Furthermore, we underscore the imperative for precise preoperative evaluation of biliary anatomy and stringent intraoperative regulation of stump length.

## Introduction

Anomalous pancreaticobiliary ductal union (APBDU) is a congenital condition characterized by the confluence of the common bile duct (CBD) and pancreatic duct occurring outside the duodenal wall. This configuration results in an abnormally lengthy common channel and the functional absence of Oddi's sphincter [1], serving as the etiological basis for congenital biliary dilatation (choledochal cyst), with which it frequently co-occurs [2]. APBDU permits reciprocal reflux between pancreatic juice and bile, fostering chronic inflammation of the biliary epithelium and bile stasis, consequently heightening the risks of cholelithiasis, pancreatitis, and biliary tract carcinogenesis [3]. Although radical surgical resection of the choledochal cyst combined with Roux-en-Y choledochojejunostomy effectively bypasses obstruction and mitigates malignant potential, this approach typically leaves the fundamental APBDU anatomy uncorrected [4]. Should an excessively long CBD remnant persist postoperatively, it may evolve into a blind loop that encourages pancreatic juice stasis, thereby facilitating long-term lithogenesis [5]. The emergence of common duct stones under these specific post-choledochojejunostomy conditions is exceptionally rare, particularly among adults. The uniqueness of the present case lies in its presentation 20 years following cholangioenterostomy. Imaging modalities revealed a P-B-type APBDU accompanied by a 2.5 cm CBD stump and a large calculus originating from the pancreatic duct. These findings illuminate the protracted risks associated with overlooked APBDU due to inadequate initial biliary imaging, compounded by insufficient shortening of the CBD stump. Clinically, this case distinctly demonstrates the dual pathogenic roles of "uncorrected APBDU anatomy" and a "surgically created excessively long blind loop," offering insights that transcend traditional pancreaticobiliary reflux theory and providing fresh perspectives on late-stage post-operative complications [6]. We employed endoscopic sphincterotomy and cholangioscopy-guided stone extraction, demonstrating that a minimally invasive endoscopic strategy can successfully manage complex scenarios while avoiding relaparotomy, thus offering valuable therapeutic reference. This report constitutes a significant contribution to the literature for two primary reasons: First, it documents a novel instance of an extremely delayed common channel stone, including pancreatic duct calculi, following cholangioenterostomy. Second, it reinforces the critical necessity of comprehensive preoperative biliary anatomical assessment utilizing advanced modalities such as magnetic resonance cholangiopancreatography (MRCP).

Third, meticulous intraoperative technical execution, particularly the precise control of the residual stump length, is critical for mitigating long-term complications [7]. Fourth, the effective management of this case establishes a pragmatic diagnostic and therapeutic algorithm for clinicians encountering analogous complex scenarios, thereby facilitating early detection, expedited intervention, and optimized patient prognoses. Consequently, comprehensive case reporting and analysis serve to augment clinical acumen, deepen pathophysiological understanding, and refine strategic approaches to diagnosis and treatment.

## Case presentation

Patient information

A 23-year-old male was admitted on November 14, 2025, presenting with a six-month history of intermittent epigastric pain that had recurred over the preceding three days. His surgical history is extensive, including prior cholecystectomy, resection of a choledochal cyst, and cholangioenterostomy performed to address gallbladder calculi, CBD stones, and the cyst itself. One year prior, an attempt at endoscopic retrograde cholangiopancreatography (ERCP) for suspected CBD lithiasis proved unsuccessful. More than a decade ago, he underwent an exploratory laparotomy to manage hemorrhage originating from the branches of the superior mesenteric artery; subsequently, 10 years ago, a stent was implanted in the portal vein to treat cavernous transformation.

Clinical findings

**Figure 1:**
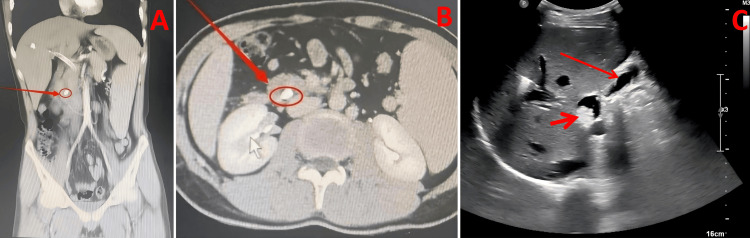
Findings from abdominal imaging examinations. Abdominal computed tomography image indicates a high-density stone shadow in the common bile duct (CBD) duodenal segment. The arrow in (A) depicts a sagittal section of the abdominal computed tomography scan, while that in (B) shows the transverse section. (C) Abdominal ultrasonography demonstrated mild intrahepatic biliary dilation (approx. 0.8 cm) (long arrow) and a hyperechoic mass measuring 1.7 × 1.5 cm with posterior acoustic shadowing in the distal CBD (short arrow).

Upon admission, vital signs were stable: temperature of 36.5°C, pulse of 78 beats per minute, respiratory rate of 18 breaths per minute, and blood pressure of 120/80 mmHg. The patient was alert and oriented, with no evidence of cutaneous or mucosal jaundice. Abdominal examination revealed a flat, soft contour with localized tenderness in the left lower quadrant, absent rebound tenderness or guarding. Neither the liver nor the spleen was palpable. Shifting dullness was not detected, bowel sounds were within normal limits, and multiple irregular surgical scars were visible on the abdominal wall. Routine laboratory parameters were unremarkable. The abdominal computed tomography imaging identified a well-circumscribed, high-density lesion approximately 1.8 cm in diameter located within the duodenal segment of the CBD. Intrahepatic bile ducts showed no significant dilation, the biliary-enteric anastomosis remained patent, and the pancreas exhibited normal morphology and density (Figure 1). Abdominal ultrasonography demonstrated mild intrahepatic biliary dilation (approx. 0.8 cm) and a hyperechoic mass measuring 1.7 × 1.5 cm with posterior acoustic shadowing in the distal CBD (Figure 1). Pancreatic echotexture and shape were normal, and the portal vein stent was correctly positioned.

Diagnostic assessment

**Figure 2:**
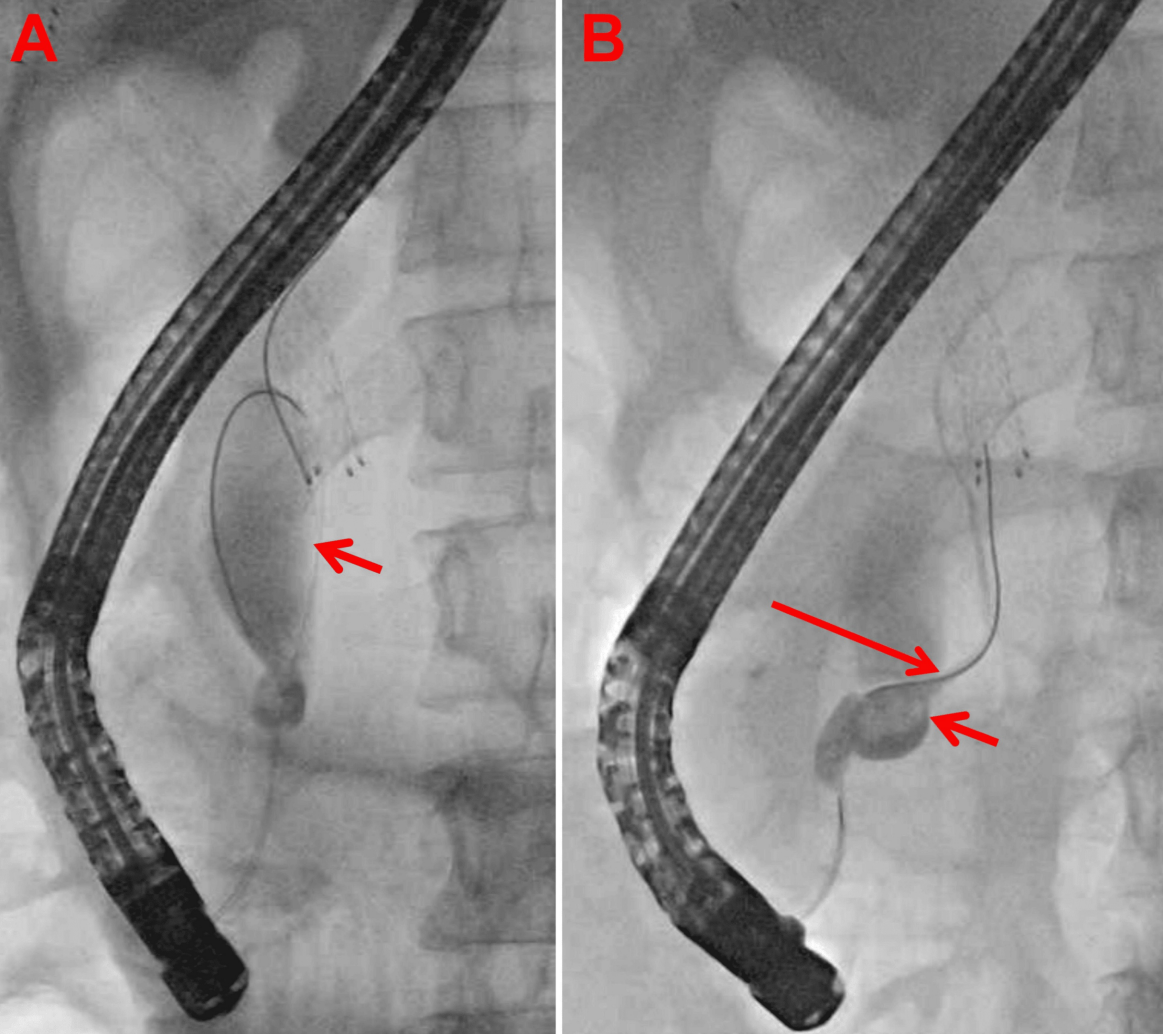
Imaging during endoscopic retrograde cholangiopancreatography. (A) The arrow indicates the elongated residual common bile duct. (B) The long arrow indicates a P-B-type anomalous pancreaticobiliary ductal union (APBDU), and the short arrow indicates the formation of stones at the confluence of the bile and pancreatic ducts.

Synthesizing the patient's clinical history, symptomatology, and radiological findings, the diagnosis was established as congenital choledochal dilation with an anomalous pancreaticobiliary duct union (P-B-type) status post-radical surgery. The residual CBD measured approximately 2.5 cm in length and harbored a calculus roughly 1.7 × 1.5 cm within the common channel. ERCP confirmed the presence of these anatomical anomalies and the stone (Figure 2). These findings suggest that the APBDU, acting as a congenital anatomical predisposition, combined with an excessively long residual CBD following radical resection, contributed synergistically to stone formation.

Treatment measures

**Figure 3:**
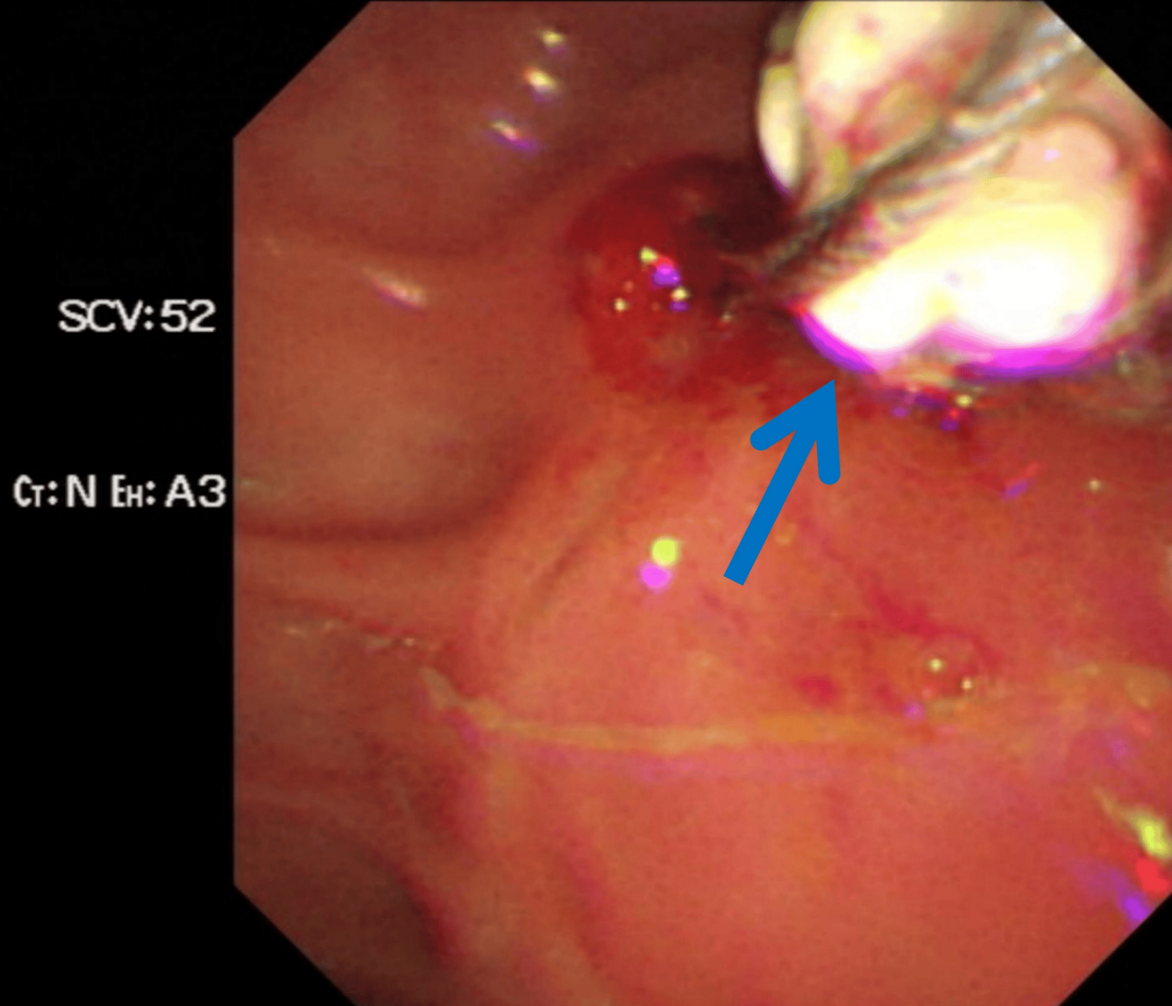
Stone extracted during endoscopic retrograde cholangiopancreatography. The arrow in the figure indicates the removed calculus, which is approximately 1.7 × 1.5 cm in size, white in color, and with a hard texture.

On November 18, 2025, the patient underwent ERCP under intravenous anesthesia. Intraoperative cholangiography confirmed the P-B-type anomalous union and an overly long residual CBD containing a tightly impacted stone in the common channel (Figure 2). As conventional lithotripsy instruments failed to extract the calculus, an endoscopic sphincterotomy extending 1.0 cm was performed. Under direct visualization via a biliary sub-endoscope, biopsy forceps were utilized to dislodge the stone and maneuver it into the residual CBD segment, where it was subsequently removed in fragments using a retrieval basket. The extracted material consisted of a hard, snow-white calculus measuring approximately 1.7 × 1.5 cm (Figure 3). An endoscopic nasobiliary drainage tube was placed prophylactically. Perioperative management included standard preoperative preparations and comprehensive postoperative care, encompassing fasting protocols and acid-suppressive therapy. Enzyme inhibition, antimicrobial therapy, and nutritional support were administered.

Follow-up and outcomes

Post-procedure, abdominal pain resolved completely within 12 hours. The nasobiliary drainage tube remained patent, with no evidence of fever, hemorrhage, or perforation. The drainage catheter was removed on postoperative day five, and the patient was discharged without complications. A telephone follow-up conducted one month after discharge revealed no recurrence of abdominal pain, fever, or jaundice; dietary intake and quality of life had returned to baseline.

## Discussion

**Figure 4:**
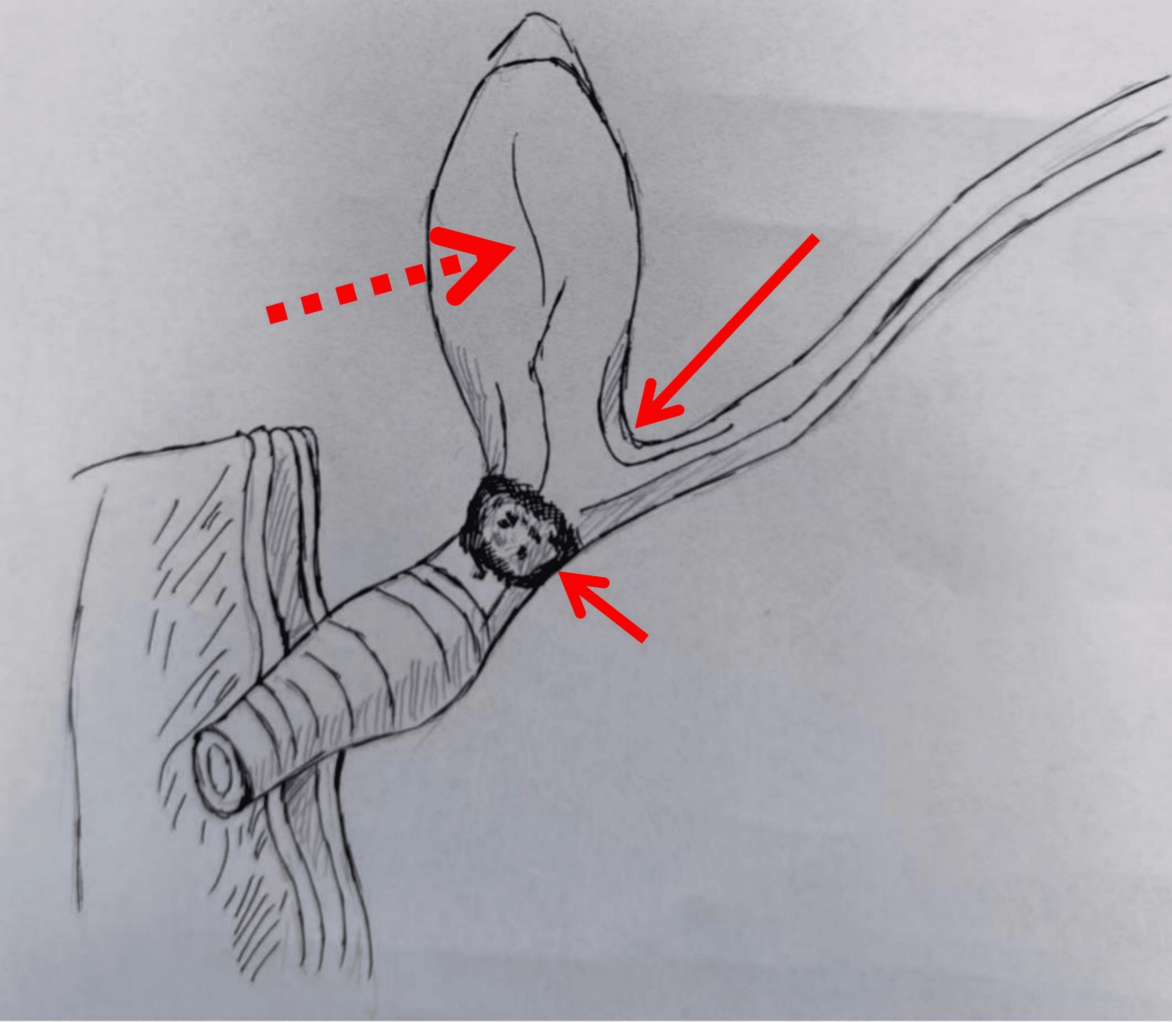
A sketch of anomalous pancreaticobiliary ductal union (APBDU) with giant pancreatic duct calculi in the common channel. The dashed arrow indicates the elongated residual common bile duct. The long, solid arrow indicates a P-B-type APBDU, and the short, solid arrow indicates the formation of stones at the confluence of the bile and pancreatic ducts. Image hand-drawn by the authors.

This case illustrates a rare delayed presentation of stones at the confluence of the bile and pancreatic ducts following biliary-enteric anastomosis in a patient with pancreaticobiliary duct disease secondary to APBDU. It is widely recognized as a primary etiological factor in pancreaticobiliary disorders, facilitating the reflux and mixing of pancreatic and biliary secretions, which induces epithelial injury and promotes stone formation [5] (Figure 4). In pediatric patients with choledochal cysts, APBDU significantly contributes to protein plug accumulation within the pancreatic duct, often preceding calculi development [8]. Distinct from most previously reported cases, our patient underwent surgical intervention postoperatively. Although the biliary-enteric anastomosis successfully alleviated biliary obstruction, it failed to correct the underlying APBDU, leaving a considerably long distal CBD stump (approximately 2.5 cm). Persistent mixing of pancreatic and biliary fluids, combined with stasis of pancreatic juice within this blind pouch, created a "double-hit" pathogenic mechanism that fostered the formation of large, hard, pancreatic-duct-type stones within the common channel [9]. This pathophysiology fundamentally differs from that of typical postoperative bile duct stones or intrahepatic calculi [10].

Further analysis indicates that, while stone recurrence after choledochojejunostomy is not infrequent, recurrent stones typically localize within the intrahepatic ducts or near the anastomotic site and are predominantly composed of bilirubin or cholesterol [9,11-13]. In contrast, stones forming within the distal bile duct stump, as observed here, can trigger recurrent pancreatitis [9]. In other scenarios, Roux-en-Y choledochojejunostomy is frequently employed as a surgical option for refractory CBD stones following multiple unsuccessful endoscopic retrievals [12]. The present case, however, highlights a delayed complication arising after prior choledochojejunostomy, suggesting that biliary diversion alone cannot fully prevent stone recurrence if APBDU remains uncorrected or if an excessively long blind pouch persists. Consequently, this case broadens the spectrum of APBDU-associated complications and underscores the critical need for comprehensive anatomical assessment and optimization of the initial surgical approach [13].

A key diagnostic challenge lies in the occult nature of APBDU and its potential omission during the primary operation. Clinical manifestations of APBDU are nonspecific and frequently mimic ordinary cholelithiasis or cholangitis, leading clinicians to overlook this condition during diagnosis [14]. Conventional imaging modalities, such as abdominal ultrasound, lack the resolution to delineate the fine anatomy of the pancreaticobiliary junction. Without preoperative high-resolution imaging - particularly MRCP - this crucial anatomical anomaly is easily missed [15]. Therefore, clinicians should routinely screen for APBDU in high-risk populations, including young individuals with unexplained cholelithiasis, patients developing gallstones after biliary surgery, and those suffering from persistent idiopathic pancreatitis [16]. MRCP serves as a noninvasive first-line screening tool capable of visualizing the length of the common channel and relevant anatomical relationships, whereas ERCP functions as both a diagnostic and therapeutic modality [17]. This case demonstrates that endoscopic interventions can be considered a first-line treatment strategy, offering both feasibility and clinical benefit to clinical decision-makers. Despite the substantial size and severe impaction of the calculi, a minimally invasive resolution was accomplished by integrating endoscopic sphincterotomy with intraductal lithotripsy and cholangioscope-guided extraction. These findings further elucidate the utility of endoscopic interventions for complex biliary and pancreatic duct lithiasis. Nevertheless, endoscopic management is not universally indicated. In scenarios involving significant common channel dilation, recurrent stone formation, unsuccessful endoscopic attempts, or suspected malignancy, surgical revision (e.g., revised choledochojejunostomy with common channel plasty) should be considered to rectify the anatomical anomalies driving stone genesis [18]. Practitioners must balance minimally invasive endoscopy against definitive surgery through rigorous imaging evaluation and risk-benefit assessment. In conclusion, this case report juxtaposes clinical data with existing literature to examine the unique pathogenesis, diagnostic challenges, and therapeutic strategies regarding rare, long-term common duct stones following choledochoenterostomy in patients with APBDU. The central tenet is that clinicians must address the underlying etiology rather than merely managing obstructive sequelae. This necessitates preoperative biliary mapping via MRCP and adjunctive modalities, alongside optimized intraoperative techniques to minimize the residual CBD stump and prevent blind pouch formation. Ultimately, lifelong surveillance is imperative for APBDU patients, even postoperatively, to ensure early detection and management of latent long-term risks.

This report acknowledges certain limitations. As a single-center case study, its findings require validation within larger prospective cohorts. Furthermore, the follow-up duration remains brief; while short-term efficacy is evident, long-term hazards such as stone recurrence and delayed complications (including biliary malignancies) warrant continuous monitoring. Future research should establish risk-stratification models incorporating anatomical classifications and surgical variables to tailor postoperative surveillance and interventions. Nonetheless, the insights gained from this case offer valuable guidance for refining the comprehensive management of similar complex cases in clinical practice.

## Conclusions

This case powerfully illustrates that uncorrected APBDU combined with a surgically created long CBD stump constitutes a significant, yet often overlooked, etiology for late-onset common channel stones post-bilioenteric anastomosis. The core clinical takeaway is the paramount importance of addressing the underlying anatomical anomaly - APBDU - during initial management, rather than merely treating its complications. Meticulous surgical technique, specifically ensuring a short CBD remnant (ideally ≤ 1 cm), is a critical modifiable factor to prevent the formation of a pathological blind pouch. Furthermore, this report reinforces the necessity of lifelong surveillance for patients with known APBDU to facilitate early detection and intervention for late sequelae.

Our findings advocate for an integrated management paradigm encompassing precise preoperative anatomical assessment with MRCP/ERCP, meticulous surgical execution to correct anatomy and minimize blind pouches, and the application of advanced, minimally invasive endoscopic techniques for treating complex complications.
